# Evaluating a Postsecondary Education Program for Students with Intellectual Disabilities: Leveraging the Parent Perspective

**DOI:** 10.1007/s10803-020-04676-0

**Published:** 2020-09-14

**Authors:** Rumi Agarwal, Laura Heron, Shanna L. Burke

**Affiliations:** 1 BRAINN Lab, School of Social Work, Robert Stempel College of Public Health and Social Work, Florida International University, 11200 S.W. 8th Street, AHC5 585, Miami, FL 33199 USA; 2 FIU Embrace, Florida International University, 11200 S.W. 8th Street, MARC 150, Miami, FL 33199 USA; 3 Department of Health Promotion and Disease Prevention, Robert Stempel College of Public Health and Social Work, Florida International University, 11200 S.W. 8th Street, Miami, FL 33199 USA; 4 Department of Industrial and Organizational Psychology, Florida International University, 11200 S.W. 8th Street, Miami, FL 33199 USA

**Keywords:** Postsecondary, Intellectual disability, Developmental disability, Parent, Evaluation, Qualitative

## Abstract

Postsecondary education (PSE) programs serving individuals with intellectual disabilities (ID) aim to improve life outcomes by increasing skills in three key areas: academics, independent living, and employment. To ensure that PSE programs are successful, ongoing evaluations are necessary. It is particularly important to gather parental perspectives given the integral role they play regarding decision making for students with ID. This qualitative study analyzed data from 58 interviews conducted with parents whose child was enrolled in a PSE program nested within a large public university. Thematic analysis with a deductive approach was the established theoretical model used to guide the analysis. Themes related to capability, opportunity, motivation, and behavior are presented, and future recommendations for PSE programs are discussed.

## Introduction

A significant employment gap exists between individuals with and without a disability. Almost 74% of individuals without disabilities are employed, compared to approximately 34% of working-age individuals with disabilities (Winsor et al. 2017). Individuals diagnosed with an intellectual disability (ID), in particular, experience even lower employment rates reported at 16% by state agencies (Hiersteiner et al. 2016). Due to these high unemployment rates, individuals with ID experience poverty, which in turn impacts community integration, social belonging, productivity, satisfaction, empowerment, independence, and quality of life (Jahoda et al. 2008; Kober and Eggleton 2005).

Evidence indicating that access to higher education is linked to increased employment rates (U.S. Bureau of Labor Statistics 2015), led to the revision of the Higher Education Opportunity Act (HEOA) in 2008, which intended to increase access to postsecondary education (PSE) programs for young adults with ID (U.S. Department of Education 2010). The HEOA increased the availability of financial aid, established the National Coordinating Center, and funded 52 Transition and Postsecondary Education Programs for Students with Intellectual Disabilities (also known as TPSIDs). TPSID programs aim to provide students with supports and services for academic and social inclusion. These include experiences that focus on academic enrichment, socialization, independent living skills, integrated work experiences, and career skills that ultimately lead to employment opportunities (Think College 2019). Five of the 52 TPSID programs have created consortiums to help fund other PSE programs for students with ID in their respective states. These efforts have driven the exponential growth of PSE programs in the United States, from 49 programs in 2008 to 270 college programs in 2019 (Think College 2019). PSE programs ultimately increase opportunities for young adults with ID to experience a college environment and increase their independent living (Miller et al. 2016), academic, and employment skills (Butler et al. 2016).

In recent years, research has been conducted on a variety of topics related to how successful or beneficial certain PSE program components are for students, such as mentoring (Culnane et al. 2016; Jones and Goble 2012), the use of natural supports (Kelley and Westling 2013), and integrating technology to enhance academic growth and independent living skills (Evmenova et al. 2017; Evmenova and Behrmann 2014; Smith et al. 2017). While evaluations of individual program components are useful for ensuring the continued success of PSE programs, holistic evaluations from the perspectives of different stakeholders are also needed.

### Leveraging Parental Perspectives

PSE programs generally collect evaluative data across several key areas including staffing, administration, student planning, student activities, employment opportunities, self-determination, and interagency collaboration. Programs may also receive feedback from the perspectives of program staff, mentors, students (enrolled and graduated), instructors, employers, and parents. Capturing parental perspectives is a crucial component of PSE program evaluations, as parents play an integral role in decision making in terms of enrolling students with ID into a PSE program, and helping students navigate through the transition stage (Culnane et al. 2016; Jones and Goble 2012).

Surprisingly, however, there is a lack of research exploring parent perspectives of a TPSID program (Sheen 2017; Yarbrough et al. 2014). In general, studies that have included parents (or families) have focused on understanding the transition of individuals with cognitive disabilities into adulthood after leaving high school (Chambers et al. 2004), or the issues families face in learning about or choosing PSE programs (Griffin et al. 2010). Research focusing on parental perspectives of PSE programs has, so far, identified what skills parents believe are important for their child to acquire during a PSE program (Sheen 2017), and program components as being most beneficial to their child with ID (Yarbrough et al. 2014). A recent study by Miller, Schleien, White, and Harrington (2018), explored desired and perceived outcomes of parents of students with intellectual and developmental disabilities (IDD) who participated in a PSE program. Findings from this study revealed that according to parents, students with IDD gained skills in several key areas including, socialization and communication, personal care, use of technology and public transportation, handling of personal affairs, and financial management.

Research has found that parental involvement is a key predictor of successful outcomes during the transition period for young adults with ID (Foley et al. 2012). Parents often find the transition period difficult, and in particular, have a hard time allowing their students to become independent in a college environment (Miller et al. 2018). By integrating parental feedback in continual program enhancements, parents will continue to see value in PSE opportunities, which will result in improved outcomes not only for their currently enrolled student but also for students who enroll in the future.

To fill this gap in the literature, the present study will address Sheen’s (2017) call for additional research by conducting a qualitative analysis of the parental experience with a TPSID-funded institution. This is particularly important, as most TPSIDs act as a model program for other PSE institutions. Using an established theoretical model to understand changes in behavior (Michie et al. 2011) and to extend prior research, feedback was gathered regarding 1) changes parents noticed in their student with ID during enrollment in a TPSID funded program, and 2) feedback related to the program overall, including an understanding of what areas of the program were helpful and which areas needed further improvement.

## Method

### Participants

Transition-aged (18–24 years old) adults with ID who apply and meet basic enrollment criteria for a TPSID-funded PSE program (nested within a large public university in South Florida) are subsequently assessed by the program psychologist as part of the admissions process. This evaluation consists of clinical interviews and a battery of assessments related to social, behavioral, and intelligence domains. Results from these assessments inform the student’s eligibility for enrollment into the program.

At orientation, parents and students are informed about ongoing data collection efforts, including an opportunity for parents to participate in an interview to provide feedback (referred to as family debriefings) at the end of the fall and spring semesters. Parents are recruited through flyers, emails, and phone calls which emphasize the voluntary nature of the data collection effort.

This study presents findings of data collected over two academic years, as family debriefings were conducted in fall 2017 (n = 12), spring 2018 (n = 10), fall 2018 (n = 22), and spring 2019 (n = 14). All parents whose child was enrolled in the program during the respective semesters were invited to participate. As such, some parents participated in more than one interview: four parents participated three times, seven parents participated twice, while all other parents participated only once over the two years. Approval from the university’s Institutional Review Board and consent forms from all parents was obtained before conducting any interviews.

Demographic data indicate that most parents who participated in the interview and shared demographic details with program staff held a high school diploma or equivalent (38%), followed by a bachelor’s degree (22%) and an associate’s degree (14%). Parents also reported having attended some college but not having a degree (10%), having a master’s degree (6%), and other levels of education (10%). The median family income was reported at $50,000 per year. The average age of the students whose parents participated in this study was 20.8 years old, and the majority were male (81%). Almost 62% of students identified themselves as Hispanic or Latino, and white (77%). In addition to an intellectual disability, just over a third of students also reported having an autism spectrum disorder (31%), and others reported having multiple disabilities (8%), speech/language impairments (27%), and other disabilities (34%). The pre-PSE program experience of most students included taking only special education classes in high school (52%), and spending most of their time in special education classes (32%), fully inclusive classes with no special education (2%), or other inclusive classes (2%).

### Data Collection

Parents who agreed to participate in the family debriefings were scheduled for one-hour structured interviews, which were conducted in-person or over-the-phone, based on the parent’s preference. In-person interviews were conducted in a private room on-campus, while over-the-phone sessions were conducted using a private conference line. Program staff were responsible for all logistics involved with contacting the parents, reserving rooms on campus, and establishing conference lines. A structured interview guide comprising 22 questions and probes designed by the PSE program was used to ensure that all program components were addressed at each interview. This study covers two academic years over which time this interview guide was piloted for the evaluation of the program. It was necessary to pilot test the instrument over the four semesters, to determine if the interview guide was able to capture changes over time among parents who participated more than once. Interviewers read out the questions and relevant probes verbatim, and parent responses were captured using detailed notes made by the interviewer. In some instances, where parents were not fluent in English, professional translators were scheduled in advance to assist with the interview session. Interviewers were master- and doctoral-level graduate research assistants (majoring in social work, public health, or psychology at the university) who worked on the PSE programs evaluation team. In any given semester, between three to six interviewers were involved. All interviewers were provided the same training on conducting interviews and collecting data, and were knowledgeable about ID, aware of challenges associated with the transition from high school to a PSE environment, and had limited contact with parents and students beyond the biannual program data collection efforts, thereby minimizing bias and enhancing rigor.

### Theoretical Framework

Qualitative studies that leverage established theories and models allow mechanisms underlying the findings to be more meaningfully understood (Meyer and Ward 2014). In this study, the COM-B change model developed by Michie et al. (2011) was used to guide our evaluation of the PSE program from the parent’s perspective. To the best of our knowledge, only two other studies have applied the COM-B model to the population of individuals with IDD (Alexander et al. 2014; Bossink et al. 2019) and no studies have utilized this model among parents of this population.

The COM-B model was developed to evaluate interventions using a “behavior system,” in which three conditions (capability, opportunity, and motivation) all interact to generate behaviors. Michie et al. (2011) define capability as “the individual's psychological and physical capacity to engage in the activity concerned”, opportunity as “all the factors that lie outside the individual that make the behavior possible or prompt it” and motivation as “all those brain processes that energize and direct behavior” (p. 4). Within the COM-B are further sub-constructs, which are defined and presented in the results section. By using the COM-B model, the present study comprehensively examined mechanisms in the environment, which contributed to changes in student behaviors related to academic, employment, and independent living as perceived by parents.

### Data Analysis

Data were analyzed using thematic analysis, which is a method used “for identifying, analyzing and reporting patterns (themes) within data” (Braun and Clarke 2006, p. 6). Specifically, interview data were analyzed using a deductive approach whereby the coding and theme development were guided by the COM-B model. Two authors read and reviewed all anonymized data collected from the structured interviews. Line-by-line coding was then conducted independently by author A using NVivo 12 software, by 'splitting' the text into smaller pieces (Saldana 2011) and assigning lines of code to the main constructs established by the COM-B model. This technique is commonly used in the health science fields when using a deductive methodology (Crabtree and Miller 1992). Author A subsequently created a tentative framework that involved analyzing each code individually to identify unique sub-constructs, such as physical opportunity and social opportunity. The sub-constructs helped pull smaller amounts of data together into more meaningful units which allowed for the development of themes (Creswell 2013).

Author B then independently coded the data using this framework, and both authors met to discuss any discrepancies until consensus was reached. To ensure the validity of data analysis and findings, this discussion involved a thorough review of themes and sub-constructs. Specifically, the authors examined each code and confirmed that they all fit under each theme and sub-construct. In addition, authors ensured that each construct accurately reflected the overall meaning of the data. A final framework was developed, which allowed author C, also familiar with qualitative methodology, to review coding decisions and provide input. By triangulating among three authors and efforts made to bracket out personal biases and experiences during coding (Creswell 2013) internal validity of the study findings was enhanced. Moreover, saturation of findings was evident with themes being repeated over the four periods of data collection. Please see Table 1 for an overview of the COM-B constructs, sub-constructs, and emergent themes.

**Table 1:** Outline of the COM-B model, sub-constructs, and key themes from interview data

Construct	Sub-Construct	Theme
Capability (C)	Psychological and Physical enablement	Mentors/faculty and program staff
		Non-academic program components
	Psychological and Physical Training and Education	Employment-related program components
		Academic opportunities
Opportunity (O)	Parent Physical and Social Enablement	Parent role, attitudes, and expectations
	Campus and Community Physical Enablement	Campus and community engagement
Motivation (M)	Reflective Education	Staying on track and direction
	Automatic Enablement	Initiative, effort, and broader horizons
	Automatic Modeling	Other peers with ID enrolled in program and neurotypical students on campus
Behavior (B)		Employment and academics
		Independent living
		Communication, socialization and overall development

## Results

This study aimed to examine the perspectives of parents related to the growth they observed in their child and programmatic changes they would recommend. As such, the criteria for improvement in all areas reported here are based upon consistent feedback received from parents who observed increased skill acquisition in their child and reported positive feedback from various program components. Results from the qualitative analysis of the interview data are presented following the four main constructs of the COM-B model: capability, opportunity, motivation, and behavior. Key themes in these four areas are reported with supporting statements from parents.

### Capability

Michie et al. (2011) distinguish between psychological and physical capability. Psychological capability refers to the ability to engage in mental processes (i.e., comprehension and reasoning), while physical capability involves having a suitable environment to promote behaviors. In the present study, capability was defined as all programmatic activities and components offered specifically to students with ID enrolled in the PSE program, which contributed to observed behaviors. Distinct themes emerged from the data that aligned with two sub-constructs identified in the COM-B model: 1) psychological and physical enablement and 2) psychological and physical training and education.

#### Psychological and Physical Enablement

Psychological and physical enablement involved specific program components that reduced barriers and offered opportunities for students to enhance their capability. The majority of parents shared that support from academic mentors offered during the program enabled their child to complete assignments on campus before returning home. Academic mentors were attributed to increased academic independence, reduced need for academic support from others, and significantly reduced reliance on parents. One parent shared that “This semester, the academic mentor has helped [student] do their work, so [student] has become more independent and doesn’t rely on us parents as much. We [parents] will help with buying materials, but we do not sit down and help, as [student] has it under control.”

In addition to academic mentors, parents reported that support from faculty mentors, professors, and program staff was instrumental in contributing to positive behavioral changes in the students. One mother shared “He [student] has many medications to take – I [mother] am no longer doing it, he is doing it. If it wasn’t for people here [program staff] giving him guidance and support, although they are sometimes stern and strict, I don’t think we would have ever broken through. Now people outside of us [the student’s parents] are assisting him and he is doing things himself. Now his blood sugar is controlled.”

Other non-academic components offered during the program such as participation in Best Buddies, Special Olympics, and social events organized for program students, were also noted by parents as being desirable, influencing independence and opening up new interests and goals. Parents, however, suggested increasing the number of planned social activities for students in the program. They shared it was important to create opportunities for students to interact and engage with others, to address the deficits in social skills commonly faced by students with ID.

#### Psychological and Physical Training and Education

Two themes emerged under this sub-construct of capability and involved program components, which serve to enhance skills and knowledge in three key areas: independent living, employment, and academics. To promote independent living skills, weekly workshops were offered by the PSE program throughout the semester, which covered topics such as transportation, money management, communication, cooking, relationships, and hygiene, among others. One parent shared, “The residential [independent living] workshops are very helpful for giving her [student] tools and increase her self-advocacy. She has also learned how to do certain things independently. She can sometimes get shy when she needs guidance, so the workshops have helped her gain confidence.” Despite significant improvements in many areas of independent living (discussed in detail later under behaviors), parents reported continued struggles with handling money. Several parents suggested additional workshops were needed to specifically address budgeting, and counting change.

Another program component repeatedly mentioned by parents was the opportunity for select students to participate in a residential experience, which allowed students to live on campus while enrolled in the PSE program. Parents overwhelmingly shared that the residential program contributed significantly to student growth. One parent stated, “The independent living program [residential program] was really amazing. It was so scary at first, you know, what is going to happen to him, will he be safe? But everything worked out, he did it and it was perfect.” Many parents whose child did not meet eligibility criteria for the residential program were disappointed that their child did not benefit from this experience and hoped that the program would consider offering it to all students in the future.

Job shadowing, internship opportunities, and support in creating resumes and conducting mock interviews were all cited as successful and useful program components, which promoted employment skills among students with ID. One parent said “The job shadowing [experience] has made him [student] more confident with what works. He did an [practice] interview and he is more confident on how to interview. He enrolled himself here [PSE program] saying he was going to be a [aquatic mammal] trainer and now he knows he has a lot of other [employment] options.” Many parents did share however, the need for more options related to internship opportunities and academic courses, to ensure that employment experiences and classes were better aligned with student interests.

Academic supports that were stated as having had a positive impact on academic skills included the availability of iPads which helped students complete iReady modules (a required interactive online instructional program) and enrollment in a public speaking class offered at the university. The latter was frequently mentioned by parents as being challenging, yet incredibly impactful. One parent shared that the “Public speaking class is really making him [student] reach further than speech therapy would in high school or seeing a speech therapist outside of school. Knowing when to speak was a struggle for him [student] before and he has now overcome that through his speech [public speaking] class and other support from the program."

Parents also made several references for utilizing methods of learning conducive for their students. For example, many parents mentioned that a visual hands-on approach was crucial to truly reinforce the skill or behavior for students with ID. In addition, repetition was of paramount importance along with consistency in the delivery. One parent stated, “He [student] is a visual learner. For example, if someone showed him how to mow the lawn or cook at home, he can learn after a few times. Whereas if he read a recipe or instructions, he would struggle more with comprehension and completion of the task.” Parents were clear that improvement in students’ knowledge acquisition, necessitated alignment of workshop and class delivery with methods of learning conducive to this population.

### Opportunity

In the COM-B model, opportunity is defined as external factors that influence behavior. Michie et al. (2011) distinguished between physical opportunity (i.e., the environment), and social opportunity (i.e., culture). In the present study, distinct themes emerged related to the construct of opportunity, which fit with the sub-constructs: 1) parent physical and social enablement and 2) campus and community physical enablement.

#### Parent Physical and Social Enablement

It was found from the findings that parents’ attitudes and expectations played a key role in shaping their child’s experiences throughout the PSE program. Many parents shared their primary goal desired from PSE enrollment was to ensure that their child gained as much independence as possible before they passed away. To support this goal, parents reported being involved in program activities as requested by program staff. For example, many parents attended meetings to stay up to date and indicated that they have grown more optimistic, and harbor greater expectations from their child with the progress they see. For example, one parent stated “He [student] is more independent now, and I [parent] trust him. He stays alone now a few nights, which would not have happened before. I have higher expectations from him than a few years ago.” Many parents also mentioned that they are less involved in the day-to-day activities of their child to encourage growth and independence. One parent explained, “I [parent] have stepped back from when he [student] first started with everything, including transportation. I have moved away from being a helicopter mom and now stepped far back. He [student] makes his own schedule. He has to learn how to do all his own things. I am preparing him for when I am not around.”

Many parents also recognized that being overprotective, fearful, and lacking confidence in their child’s ability, acted as barriers to their growth and independence. One parent stated, “He [student] has improved a lot and taken this [independent living] to a whole other level. He can take an Uber from [name of town] to downtown and can navigate really well. Before he could not even cross the street. I think this may be because I [parent] did not let him, but now he is very independent.” Another parent shared a similar sentiment, “Managing money is very difficult [for student]. But I also realize that I need to allow him to take on more responsibility with managing money, but I [parent] have not let him. But now I need to let him learn and take responsibility and learn to manage.”

While some parents consciously chose to step back from being deeply involved in their child’s academic life, a few parents remained concerned about the lack of feedback regarding student’s progress in the program. Parental attitude regarding the need for communication and program updates, ranged from being a necessary program component to being appreciated if offered, to not necessary at all. For example, one parent voiced that, “We [parents] want a constant update because we don’t know if our daughter is getting anything out of this [program]. Maybe a weekly or monthly email with important dates like a calendar [would be helpful]? Just definitely [need] more communication because our daughter says she is doing good but how do we know if she really is?” Other parents held strong opinions about wanting to get a report about their child’s academic test scores. However, other parents explained that feedback was not necessary, as it meant that their child was becoming more independent and thus achieving desired goals. One parent stated that she “Was in the dark about program information often, but that may be because “he [student] thinks that now that he is a college student, he doesn’t have to involve us, and I [parent] guess that’s a good thing.”

#### Campus and Community Physical Enablement

This sub-construct of opportunity refers to how student’s access to and involvement in campus, clubs, activities, organizations, or events offered both on-campus and in the community, influenced student behavior. Parents agreed that the opportunity for their child to experience a large university campus like a “typical college student” was one of the most rewarding experiences for them and their child. Most parents shared that their student was more social, participated in more activities outside of their interest, and overall, were more independent as a result of campus experiences. One parent shared that “The school [university] is big so when he [student] navigates campus it teaches him to not be afraid of walking alone or being independent because he has learned to figure out those situations. For example, once he was dropped off by STS [Special Transportation Service] at the wrong door and he had to find his group on his own and it actually helped him a lot.” Several parents also mentioned that their student was now more engaged in the community, participating in church activities, and undertaking volunteer work.

### Motivation

Within the COM-B model, motivation refers to the processes in the brain which direct behavior, beyond conscious decision making, which includes reflective motivation (i.e., the processes which involve evaluations and plans) and automatic processes (i.e., emotions and impulses; Michie et al. 2011). Distinct themes emerged from the data aligning with the following three sub-constructs: 1) reflective education, 2) automatic enablement, and 3) automatic modeling, which all relate to a student’s motivation that ultimately impacts their behavior.

#### Reflective Education

Reflective education refers to the process of providing students enrolled in the PSE program with goals that aim to provide a sense of direction and help students stay on track. All students in the PSE program participated in a Students Transitioning to Adult Roles Person-Centered Plan (STAR PCP), which allows students to set goals in the following areas: career development and employment, academic enrichment, campus and community engagement, independent living, and self-determination (Hayes and Muldoon 2013). A STAR PCP meeting is held with each student, along with family members and program staff during the first academic semester to encourage students to set goals for themselves. Many parents shared that the STAR PCP meeting provided students with a visual of their overall goals, which motivated and helped them to stay focused throughout the program. One parent stated that “Prior to the program, when you asked him [student] what he wanted to do, he had no answer. Now he has specific goals and he wants to get steady employment, [and] learn to drive. These are things that 6 months ago were not on his radar.” Another parent shared that “He [student] now has defined goals: he says he has a Plan A and Plan B and I [parent] like that he now has goals.”

#### Automatic Enablement

Having clear goals and direction, allowed students to engage in automatic enablement, defined as showing initiative, effort, and broadening one’s horizons. Parents noticed that students were beginning to take more initiative, making effort to learn, and increasingly taking responsibility for helping with chores at home such as cooking and laundry. One parent expressed “She [student] cooked rice for the first time last week. She wanted to do it and she did,” while another parent shared that “He [student] now takes it upon himself to do things like doing the laundry for the family without being asked or told.” Parents also frequently mentioned that their student was now open to broader employment options because of the variety of internship and job shadowing opportunities offered by the program. For example, one parent shared that her student “Has become more open about employment options with his experience in the diverse internship positions he has been placed in. I [parent] am grateful that he [student] is getting internships in areas outside of the area he had thought about as it broadens his confidence in his ability to do other jobs.”

#### Automatic Modeling

The subconstruct of motivation, automatic modeling, refers to how peers with ID in the program and other neurotypical students on campus motivate students to engage in certain behaviors. Parents mentioned that both peers and neurotypical students acted as role models, which prompted student’s motivation to graduate, attain employment, and live independently. Many parents shared similar sentiments as stated by the following parent, “Since he [student] is friends with a good group of students, who are goal-focused, he is also motivated to follow them [peers with ID]" while another parent shared that his child had a friend who is graduating soon, which contributed to the students desire to have a paid internship, job and earn money as well.

Findings also illustrated that peers played a crucial role in enhancing student participation in social events, improving confidence to express thoughts and opinions, ask peers for help and find solutions independently without the involvement of parents. For example, one parent shared that “The program has given him [student] a platform to be able to communicate with peers better. He has a core group of students that give him the ability to express his identity and self.”

### Behavior

Using the COM-B model, this study examined what behaviors students were engaging in, because of improved capability, opportunity, and motivation. Three themes emerged which align with programmatic goals of increasing student 1) employment and academic achievement, 2) independent living growth, and 3) socialization, communication, and overall development.

#### Employment and Academic Achievement

Almost all parents reported that students were more independent with assignments and homework and less likely to ask for help, as they completed homework on campus before coming home. In addition, significant improvements in reading and writing abilities were reported by parents. Many parents agreed that their children could express themselves better through writing than speaking. They shared that improvements in writing skills were apparent from the text messages they received from their child. For example, one parent shared “I see that [improvement with writing] with his text messages and communication. His vocabulary has improved too.” Parents, however, conveyed that handling money was a necessary life skill in which observable growth was lacking. One parent explicitly shared that “This type of math [money management] is important, but not so much algebra.” Several parents highlighted the need for money management workshops, which focused on practical skills rather than mathematical concepts that students with ID may not utilize in the future.

As mentioned previously, parents shared that students appeared to be more open to alternative employment options, have more clarity on their employment interests, and more stable goals. One parent shared that “My son has increased awareness of being on task and a sense of independence in the workforce.[He] understands social and work roles more clearly.”

#### Independent Living

The majority of parents noted improvement in behaviors related to independent living skills. Students were generally more responsible with managing time as observed by arriving to class and other commitments as scheduled. One parent shared that “He [student] knows that he does not like to be late, he knows that he wants to be at the place 15 min before the event begins. This is new and unusual for him as he normally ran on his own time schedule.” Another parent shared “He [student] has become more diligent with his time and has become more responsible with time management. He has learnt this through the program. He reminds his dad now that it’s time to leave or time to take a break.”

Students’ ability to cook and clean improved since being enrolled in the program. Parents who were hesitant to allow their child to work in the kitchen expressed greater confidence in their child’s ability to cook safely “He [student] cooks on his own now and I [mother] am no longer scared that he will hurt himself or be in danger. He is more aware of dangerous things and more careful and conscious when taking on tasks.” Another parent expressed that her son “Can make food for himself and do laundry. On days I [mother] have been late at work, he has made himself a meal in the microwave and fed the cat. I have seen a great improvement.”

Students exhibited growth in other areas, such as doing dishes, loading or unloading the dishwasher, and keeping their room clean. In addition, students had become more aware of their self-presentation, which is important in employment settings. For example, one parent shared that her son now “Knows the difference in clothing for an interview and dressing for school.” Students were also utilizing transportation more independently. With Uber and Lyft applications available on student phones and bus schedules available online, students were able to plan their day independently and engage in social activities. For example, one parent shared “He [student] has learnt the Uber application, which brings you into the community. The guardianship would have limited him in that we [his parents] would have had to be contacted for him to leave campus. He Ubers to [town] to go to a card shop he likes and also went to a convention by the airport. He is now able to get himself into the community, when before he was limited by what we [his parents] were able to do for him. I never thought he would get to that point. I feel that the entire program has contributed to this.”

#### Communication, Socialization and Overall Development

Although the overall goals of the PSE program are to enhance independent living, academic, and employment skills, it is equally important that students with ID enhance skills in areas such as communication, socialization, and self-advocacy. Thus, parent identification of growth in these areas is particularly important. For example, one parent expressed that her daughter is “Much more social now. Before, she [student] would only say like three words to a stranger before, but now she will speak much more openly to everyone. You saw when you met her today. Her verbal and interviewing skills have improved so much. She wouldn’t be able to do that without [PSE program] help.”

Another parent shared an example demonstrating how advocacy skills have improved, “He [student] was never one who was comfortable going to someone in authority and now he realizes it is beneficial to him to be able to express himself when he feels he’s not being understood or heard. Recently he missed a class or appointment and was almost about to get in trouble. But when he went to the meeting, there was a rally on campus – they [the staff] thought he just didn’t show up and he didn’t go through the proper channels to let them know. He tried to explain but it didn’t go through the proper channels. He called me [parent] upset, and I said *he* had to take care of it. A couple days later, I found out he went to [program director and program manager’s] office to address the issue. He would probably not have done this before. I believe it is the residential workshops [independent living workshops] on different subjects that have helped. All of the students are together with the adults who facilitate. It’s almost like there’s a referee in the room and students can voice their thoughts.” Other parents also stated, “He goes up to the teachers and expresses himself without us having to advocate for him. He is not fearful anymore to approach the professor and get the help he needs.” Overall, parents observed enhanced levels of maturity, confidence, self-esteem, advocacy, socialization, and a sense of responsibility.

### Overall Parent Perspectives

Although parents suggested some areas for improvement within the program (mentioned previously), parents appeared to value and perceive the PSE program positively overall. Many parents shared sentiments similar to one parent who stated that “Everything has worked out so well and I have seen so many benefits already. I see all of the students succeeding – they are happy in the college environment and are proud of themselves.” Another parent shared “I [parent] always thought he [student] would be very dependent on me. He will always need some type of supervision, but now I feel he would be more prepared to take care of himself if anything happened to us” with one other parent stating “Everything is going perfectly. I thought it would take at least one year for avenues to open up for the future, but the program is already offering him the right path.”

## Discussion

This study used the COM-B model (Michie et al. 2011) to guide the evaluation of a TPSID program serving students with ID, from the perspective of parents. Parents play a central role in the life trajectory of their child with ID and are therefore influential predictors of successful outcomes from the transition period after high school (Foley et al. 2012). As such, gathering parental feedback to improve PSE programs is imperative. Findings from this study built upon existing literature by holistically examining parental perspectives of their student’s improvements and challenges, as well as areas of the program that were beneficial, and those that need improvement.

### Student Improvement and Key Challenges

Across the two years of data collection, parents’ observations and feedback allowed an insight into areas of growth related to skills and abilities. Based on consistent parent reports, it was evident that students with ID improved in several important areas relating to academics, independent living, and employment, among others. For example, students showed increased reading and writing ability and relied less on their parents for help completing homework. However, most parents reported that math and money management were persistent challenge areas among students with ID. Parents did report increases in levels of independence, so much so that some were more comfortable leaving their child home alone. Students also demonstrated growth in skills related to cooking, cleaning, personal care, time management, and using public transportation. These findings are consistent with prior research where parents also reported increased use of public transportation and the ability to manage personal care (Miller et al. 2018).

Most students were also more open to broader employment options, were more aware of appropriate self-presentation for different occasions, and improved their verbal and interview skills. These are all significant areas of growth related to employment success. Students also improved in communication and socialization skills as a result of being immersed in the college environment and interacting with both peers with ID and neurotypical students. Finally, parents reported that students demonstrated overwhelming growth in maturity, confidence, responsibility, and self-advocacy—all crucial areas of personal development that support the life trajectory of a student with ID.

### Most Beneficial Program Components

Participation in the residential program, independent living workshops, inclusive academic classes, and having access to academic and faculty mentors and supportive program staff were all commonly referenced by parents as being valuable and effective in helping their child grow. Similar to findings from previous studies, employment experiences offered through job shadowing and internships (Griffin et al. 2010) and the opportunity to participate in large campus and engage as a typical student would (Yarbrough et al. 2014), were stressed upon as being valuable and essential for student growth. Interestingly, the child’s safety on campus was noted as one of the most important factors in considering PSE options in the study by Griffin et al. (2010). However, in this study, only a few parents expressed concern for safety concerning cooking, and only one parent shared safety concerns related to participation in the residential program. The limited mention related to campus safety may be a reflection of the parents' confidence in the program staff to provide a safe campus environment for the students.

Having access to resources such as an iPad was useful in helping the student’s complete coursework, and public speaking classes were commonly cited as being beneficial in increasing communication skills. External experiences such as engaging in Special Olympics were also regarded as influential predictors of independence among students with ID. Parents also shared that the STAR PCP meeting and having visual goals were beneficial in helping students stay on track throughout the program.

### Areas for Improvement

Although many parents were satisfied with the PSE program, some desired greater variety in academic coursework and internship opportunities to ensure alignment with student interests. A few parents, however, contradicted this perspective and valued that their student was being exposed to experiences outside their established areas of interests, which seemed to broaden horizons and allow for the consideration of new possibilities. Thus, this opposing sentiment reveals the need for programs to further evaluate if providing additional variety to students would be in their best interest. Additional workshops related to specific challenge areas such as math (money management) are also necessary. Parents stated that using a hands-on visual approach to learning would be the most beneficial to ensure knowledge and skill gains.

### Parent’s Role

Findings from this qualitative analysis demonstrated that some parents had been holding their child back due to their fears and expectations of what their child could accomplish. At the time of enrollment into the PSE program, parents are encouraged to allow their students to be more independent, and let the student take on more responsibility in areas such as managing their schedules, attending classes and internships, and completing work on time. Allowing students to take responsibility and make choices is a desired expectation that many young adults with disabilities have of their parents (Cooney 2002). In line with prior research, the present findings indicated that as parents saw their child grow, and the potential for self-sustainability and independence in the future became more evident, parental expectations for their child’s future increased (Doren et al. 2012). As a result, some parents felt more comfortable taking a step back. These findings speak to the importance of not underestimating the abilities of young adults with ID and the value of PSE programs to help parents realize the many possibilities that are available to their child (Yarbrough et al. 2014).

While many parents agreed that independence was an important goal of the PSE program, there was a wide variety of opinions regarding communication between the program and parents. Some parents were comfortable with receiving limited feedback, while others voiced the need for greater involvement and constant feedback regarding their child’s performance in the program. This finding is in line with previous literature, where many parents of young adults with disabilities feel the need to continue to protect their “vulnerable” child (Gillan and Coughlan 2010), and remain as their “safety net” while paradoxically hoping to be less involved in their child’s life (Cooney 2002; Martinez et al. 2012) and encourage independence (Bianco et al. 2009).

PSE programs, however, are bound by the Family Education Rights and Privacy Act (FERPA; FERPA Regulations 2011). FERPA guidelines prohibit the sharing of educational records once a student is 18 and older, regardless of disability. As a result, there remains a challenge between parental expectations and the program’s legal obligations which is unique to PSE settings and parents of children with disabilities (deFur et al. 2001). Looking forward, programs may want to consider sharing general programmatic information with parents at regular intervals regarding activities and topics of workshops. This may augment student learning through consistent messages shared at home and the college environment.

This study highlights the delicate balance that PSE programs have to address by providing students with ID a supportive environment for cultivating independence and growth, while also reassuring parents and setting meaningful boundaries, which enhance (rather than deter) shared parental, student and programmatic goals.

### Limitations

Although efforts were made to ensure rigor in data collection and analysis, it is important to recognize a few study limitations. First, multiple interviewers and translators were involved in administering the interviews, and recording devices were not used. Instead, detailed notes were taken by the interviewer, and where possible verbatim quotes were captured. Therefore, even though all interviewers received the same training, it is possible that responses were still affected by interpretation, language, and social desirability bias. Future research should look to further standardize the interview process and also use voice recorders to enhance the validity of the data. Another study limitation was the small sample size, and overall low participant rate per semester, which could indicate volunteer bias. As a result, it is possible that findings may not generalize across all parents of students in the program and could instead, reflect the opinions of a few parents who were more involved or more likely to notice changes in their child. Future studies should build on these findings by encouraging participation from a wider range of parents, perhaps through the use of incentives.

Given the voluntary nature of parent participation in the PSE parent debriefings, and pilot efforts regarding data collection efforts in general, the lack of demographic data for parents is another study limitation. While there was limited in-depth parent data, students whose parents participated in the interviews and had demographic data on file represented a homogenous group regarding race and ethnicity, and it is important to note that the study findings pertain specifically to a PSE program in the Southeastern United States. Therefore, findings may not generalize to other parent populations who have children in similar PSE programs across the country. Future studies should expand upon these findings by examining PSE programs in other regions of the country from the parental perspective, and also by collecting more demographic data on parents.

## Conclusion

PSE programs serving students with ID aim to enhance quality of life through gains in academic achievement, employment, and independent living. Conducting thorough evaluations of such programs is essential to ensure continuous improvement and maximize the outcomes for individuals with ID and their families. The present study demonstrated the value of gathering input from parents regarding areas where the program can improve and where their child is demonstrating the most growth. Ultimately, parents must be given a voice in the ongoing improvement of these programs, as they are influential caregivers whose decisions influence the trajectory of the student with ID.
